# Semi-classical Monte Carlo algorithm for the simulation of X-ray grating interferometry

**DOI:** 10.1038/s41598-022-05965-7

**Published:** 2022-02-15

**Authors:** Stefan Tessarini, Michael Karl Fix, Peter Manser, Werner Volken, Daniel Frei, Lorenzo Mercolli, Marco Stampanoni

**Affiliations:** 1 Institute for Biomedical Engineering, University and ETH Zürich, 8092 Zürich, Switzerland; 2 Swiss Light Source, Paul Scherrer Institut, 5232 Villigen, Switzerland; 3 Division of Medical Radiation Physics and Department of Radiation Oncology, Inselspital University Hospital Bern and University of Bern, Bern, Switzerland; 4 Federal Office of Public Health FOPH, Schwarzenburgstrasse 157, 3003 Bern, Switzerland; 5 Laboratory for High Energy Physics, University of Bern, Sidlerstrasse 5, 3011 Bern, Switzerland

**Keywords:** Biomedical engineering, Software, X-rays, Imaging and sensing

## Abstract

Traditional simulation techniques such as wave optics methods and Monte Carlo (MC) particle transport cannot model both interference and inelastic scattering phenomena within one framework. Based on the rules of quantum mechanics to calculate probabilities, we propose a new semi-classical MC algorithm for efficient and simultaneous modeling of scattering and interference processes. The similarities to MC particle transport allow the implementation as a flexible c++ object oriented extension of EGSnrc—a well-established MC toolkit. In addition to previously proposed Huygens principle based transport through optics components, new variance reduction techniques for the transport through gratings are presented as transport options to achieve the required improvement in speed and memory costs necessary for an efficient exploration (system design—dose estimations) of the medical implementation of X-ray grating interferometry (GI), an emerging imaging technique currently subject of tremendous efforts towards clinical translation. The feasibility of simulation of interference effects is confirmed in four academic cases and an experimental table-top GI setup. Comparison with conventional MC transport show that deposited energy features of EGSnrc are conserved.

## Introduction

Phase sensitive X-ray imaging techniques^1–3^ such as propagation based imaging^4^, grating interferometry^5–9^, speckle based imaging^10,11^, or edge-illumination^12^ provide complementary contrasts in addition to absorption contrast. In Talbot-Lau X-ray grating interferometry^8^ (GI), for example, an absorption and a phase grating are inserted into an incoherent X-ray beam to generate interference patterns on an imaging detector, from which absorption, differential-phase, and dark-field contrasts are retrieved. GI has great potential for application in material science, biology and medicine. The latter two mainly due to enhanced soft tissue visibility^13,14^. For clinical prototype development, simulations are of great value to estimate different performance and patient relevant quantities like radiation dose, beam spectra, fringe visibility, and scattering contributions. However, due to occurrence of inelastic (Compton and photo effect) scattering and interference phenomena the well-established simulation techniques like wave propagation^15,16^ and Monte Carlo (MC) particle transport^17^ are not sufficient to cover all relevant quantities. While MC particle transport is the gold standard for the estimation of scattering related quantities in fields like X-ray absorption imaging and radiotherapy^18^, it can not simulate interference phenomena. Wave propagation, on the other hand, which is used for the simulation of the interference patterns in phase contrast imaging^19,20^, can not simulate inelastic scattering events explicitly, which makes it unfeasible for scattering related quantities like radiation dose. Furthermore, wave propagation is often difficult to use in more complex situations, e.g. when a biological sample is inserted into a grating interferometer.

Previous MC approaches to the simulation of phase sensitive X-ray imaging setups include combinations of MC with wave propagators^21–23^, MC that imitate the Huygens-Fresnel principle^24–27^, and pure ray-tracing algorithms^28,29^. However, the algorithms applicable to GI either suffer from long computation times unfeasible for clinical prototype simulations or consist of a combination of more than one simulation technique, which is physically unsatisfactory.

Aiming for a MC framework for simulation of interference and scattering based quantities in GI with the potential scalability to clinical volumes a new MC algorithm is developed based on basic quantum mechanical concepts, e.g., indistinguishability of paths for a photon to arrive on the detector, combined with classical approximations. The MC algorithm for the calculation of the expected detector signal in a GI setup is built in two steps, as described in the Methods section. First a pure ray tracing algorithm for the computation of the interference pattern without scattering is developed. Afterwards explicit simulation of inelastic and Rayleigh scattering events are introduced into the ray-tracing algorithm. The resulting MC algorithm describes scattering and interference phenomena in one framework and was implemented as an object oriented user friendly easy to expand extension library of the well-established EGSnrc^30^ MC particle transport code. Apart from uniform splitting at gratings that imitates Huygens principle similar to previous approaches, new variance reduction techniques for fast transport through flat (planar) absorption and phase gratings are introduced to address the scalability for the simulation of clinical GI prototypes. Although this work focuses on X-ray Talbot-Lau interferometry and aims at the simulation medical applications, the simulation framework is not restricted to grating based X-ray applications. Relying on c++ inheritance new optics components can be added to the framework and directly used by the provided interferometer class.

## Results and discussion

The feasibility of the algorithm and the variance reduction techniques to model interference and scattering phenomena is demonstrated with five examples motivated by but not exclusive of X-ray Talbot–Lau interferometry. Each example focuses on a different aspects required for the simulation of GI setups, such as basic interference patterns, absorption and differential phase imaging, deposited energy and the visibility in a laboratory GI setup. At first the capability of the algorithm to simulate basic interference patterns is validated by simulations of the double-slit experiment and the Talbot carpet for phase gratings. Since largest improvements in computation time are expected for transport through phase gratings, the example of the Talbot carpet serves as benchmarking case for the performance of Fourier splitting compared to Huygens splitting. The compatibility with simulation of scattering effects is demonstrated by comparison of the deposited energy with a modified tutor2pp EGSnrc user code. Finally, the visibility in a table top laboratory setup using a polychromatic source is simulated and compared to experiment. In all simulations the standard EGSnrc cross section data file 521ICRU is used for simulation and validation. The real part of the decrement of the refractive index are taken from the CXRO online X-ray database^31^ for an energy of 17 keV and interpolated for other wavelengths assuming a $\lambda ^2$-dependence^32^. Two linux systems with an Intel Xeon E3 and an AMD Ryzen 7 3700X, respectively, were available for simulation.

### Basic interference phenomena

#### Double-slit experiment

**Figure 1:**
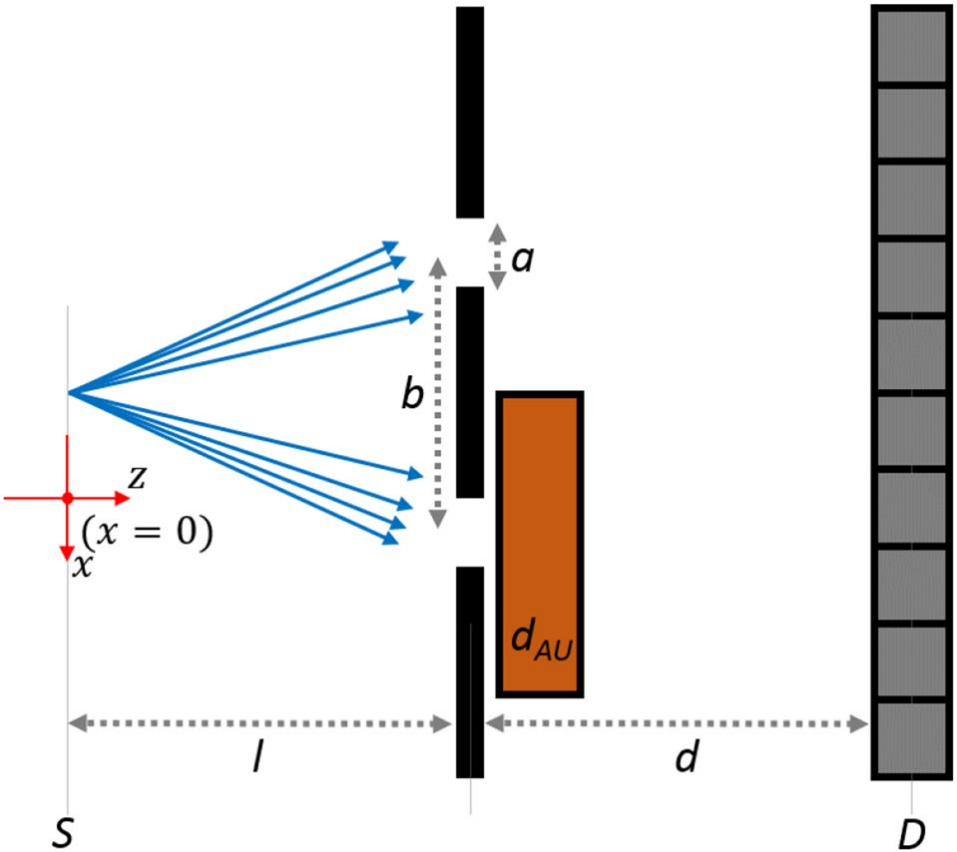
Simulation setup for the double-slit experiment. A monochromatic point source is focused on two slits of a grating with a slit width $a = 0.2\,\upmu \text {m}$ and a distance $b=2.2\, \upmu \text {m}$ between the slits. In one simulation a gold block with thickness $d_{\text {AU}} = 0.1$ mm (>99.9% absorption) is placed after one of the slits. The distance from source to the slits $l=110$ cm and the distance between the slits and the detector $d = 10$ cm.

**Figure 2:**
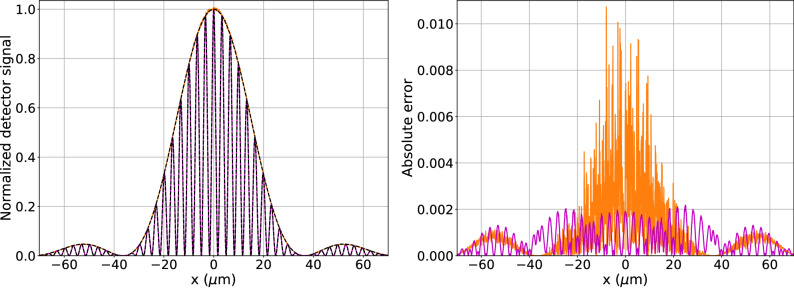
Double slit and blocked double slit interference patterns on the left. The MC simulated profiles of the double slit signal in magenta, the blocked double slit in orange and the analytical intensities for double and single slits in black. The MC signals were divided by the number of paths squared for each data point. Furthermore, the results were normalized to equal area under the curve. For convenience the off-axis single slit signal is shifted to the center. The absolute difference between the theoretical and the MC signals is shown on the right, for the double slit in magenta and blocked double slit in orange.

The first example includes two versions of the double-slit experiment in Fig. 1 validating the feasibility for the simulation of basic interference phenomena. First a standard double-slit experiment is simulated. The second simulation includes a 0.1 mm thick gold slab blocking the beam 10 $\upmu$m behind one slit. In both cases the optical element implemented for source gratings using Fourier splitting is used with a 17 keV coherent point source generating $10^5$ coherent histories, collimated onto two slits of $a = 0.2\,\upmu \text {m}$ with a separation of $b=2.2\, \upmu \text {m}$. The simulations were performed as a single task on the AMD core with computation times of roughly 260 s for the double-slit experiment and 670 s for the blocked case. To reduce the impact of fluctuations of the number of paths, the resulting raw MC output was renormalized by the number of paths squared for each data point. The resulting MC signals shown if Fig. 2, agree well with analytical results with a root mean squared error (rmse) of 0.0009 for the double slit and 0.004 for the blocked double slit compared to the analytical single slit signal.

### Talbot carpet

The spatial dependence of the interference patterns and the performance improvement promoted by Fourier splitting over Huygens splitting is investigated by means of the Talbot carpet for a plane wave source of 20 keV passing a flat $\pi$-phase grating with 4 $\upmu$m period and duty cycle of 0.5. For performance comparison of the two splitting modes the signal at 10 grating-to-detector distances in the Talbot carpet are compared to a Fresnel propagator based Fourier optics simulation^15^, for different splitting numbers $N_{G1}$ and number of histories. Huygens splitting is tested with splitting numbers $N_{G1}= 225, 450, \dots  14400$ (doubling each time) and number of histories $N_{\text{H}}$ equal to $5\times 10^5$ and $10^6$. For Fourier splitting the parameters $N_{G1} = 5,7,\dots , 41$ (Eq. (25)) are combined with $10^6$, $4\times 10^6$, and $8\times 10^6$ histories to compensate for the lower number of paths propagated compared to Huygens splitting. In a second step the entire Talbot carpet is simulated for the best Fourier splitting case with $N_{G1} = 41$ and $8\times 10^6$ histories and compared to the wave propagation result.

**Figure 3:**
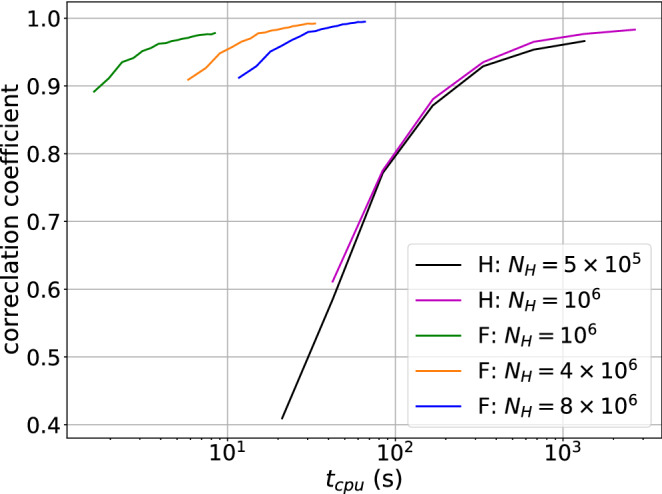
The Pearson correlation coefficient of the MC generated signals and the wave propagation signals for different number of histories and splitting numbers. Each correlation coefficient is computed from 10 grating-to-detector distances. The curves represent the two implemented splitting procedures, Fourier and Huygens splitting, for fixed number of histories, as indicated by the legend. For both splitting procedures the average simulation time (horizontal axis) has a roughly linear relationship with splitting numbers $N_{G1}$. For improved readability the simulation times are shown in logarithmic scale.

The simulation quality for each parameter pair ($N_{G1}$ and $N_{\text{H}}$) is assessed by the Pearson correlation coefficient between MC and wave propagation for the 10 detector signals and plotted against the average simulation time in Fig. 3. Each curve in Fig. 3 represents simulations with a specific splitting method with a constant number of histories. For improved readability, due to the large time differences the time axis is logarithmic. For both splitting methods the simulation time is roughly proportional to the number of histories and splitting numbers, which leads to a consistent large benefit in terms of simulation time for Fourier splitting over Huygens splitting when comparing simulation results with similar correlation coefficient. For instance, the best Huygens splitting simulation (highest correlation coefficient) is a factor of 74 to 136 slower than Fourier splitting simulations with similar correlation coefficient, as indicated by Table 1. Additionally, the correlation coefficients in Fig. 3 show a convergence towards the wave propagation signal for both splitting approaches, which is sufficient for most MC applications, especially in cases where the interference pattern can not be measured directly and an average over several periods is acquired in a phase stepping curve.

**Figure 4:**
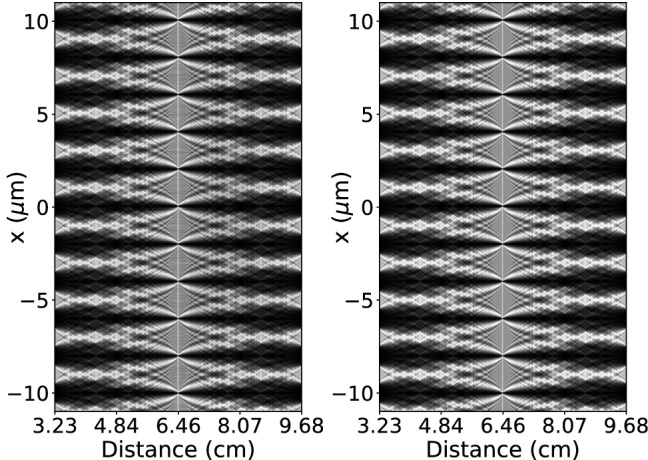
Talbot carpets between the first two fractional Talbot distances at 3.23 and 9.68 cm obtained with MC on the left and with a wave propagator on the right for comparison. Grating-to-detector distance is plotted on the horizontal axis. The vertical axis corresponds to the position x on the detector.

The Talbot carpet for $N_{G1} = 41$ Fourier splitting and $8\times 10^6$ histories is shown in Fig. 4 alongside with a wave propagation simulated carpet. For the full carpet the average simulation time per detector distance was 68 s, resulting in a correlation coefficient of 0.9913 and a root mean squared error of 0.04. As shown in Fig. 3 the simulation time can be reduced significantly dependent on the demands on simulation quality for the respective simulation task.

**Table 1:** Correlation coefficients and average simulation times for the best (highest correlation coefficient) Huygens splitting case and the Fourier splitting simulations leading to similar quality.

Splitting mode	Huygens	Fourier	Fourier
$N_H$	$10^6$	$4 \times 10^6$	$8\times 10^6$
$N_{G1}$	14,400	23	21
Correlation coefficient	0.9831	0.9828	0.9838
average simulation time	2682 s	20 s	36 s

### Absorption and differential phase

**Figure 5:**
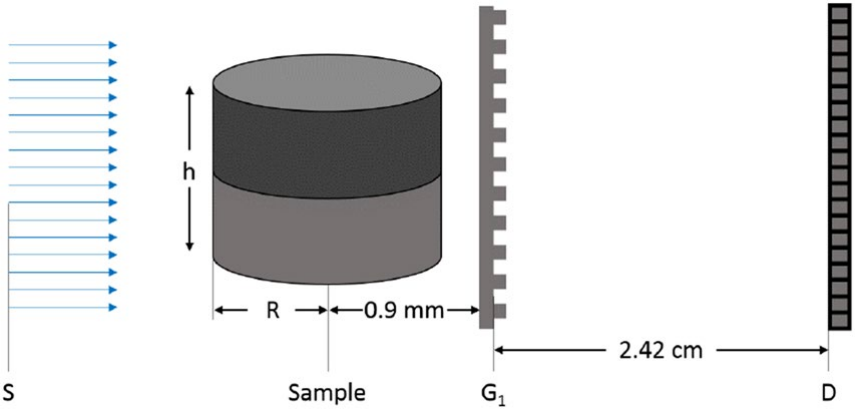
Simulation setup for the absorption and differential phase projections of a cylinder with radius $R=0.87\,\text {mm}$ and height $h=0.9\,\text {mm}$. The cylinder consists of a polystyrene part and a silicon part, sketched with different gray values. The 20 keV plane wave is simulated by starting parallel paths from a source plane S. The detector is placed at the third fractional Talbot distance of (2.42 cm) from the grating.

**Figure 6:**
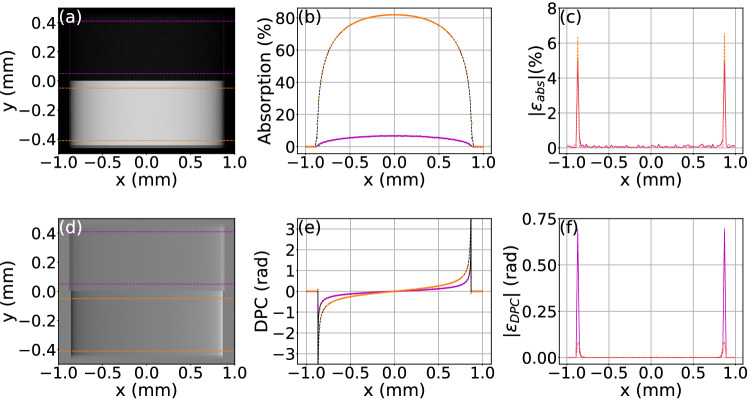
Absorption and differential phase signals of a cylinder. Retrieved absorption (**a**) and DPC (**d**) projections. Lines indicate regions for the averaging of profiles. (**b**) MC simulated averaged absorption profiles of Silicon (orange) and polystyrene (magenta) and the analytically calculated signal using Beer-Lambert law (black). (**e**) MC DPC profiles for Silicon (orange) and polystyrene (magenta) and the corresponding analytical signal^9^ (black). (**c**) and (**f**) Absolute difference between MC and theoretical signals.

The feasibility to model absorption and differential phase imaging modalities of GI is demonstrated by the simulation of a projection of a cylinder Fig. (5) with a radius of 0.87 mm and a height of 0.9 mm consisting of an upper polystyrene part and a lower Silicon part. The simulation setup consists of a 20 keV coherent plane wave illuminating a $\pi$-phase grating with 2 $\upmu$m period and a duty cycle of 0.5 and a detector at third fractional Talbot distance. The reference and sample image simulations were performed on the AMD core with $5\times 10^8$ coherent histories and a Fourier splitting of $N_{G1} = 5$, with a resulting simulation time of 18 min for the reference and 24 min for the sample simulation. The resulting absorption and differential phase contrast (DPC) projections are shown in Fig. 6a,d, respectively, next to a comparison of the averaged line profiles in Fig. 6b,e to analytically calculated profiles and the absolute difference between MC and theory in Fig. 6c,f. Both cases (absorption and DPC) show good agreement between simulation and theory apart from the border regions of the cylinder. The rmse of the absorption profiles shown in Fig. 6b are 0.0091 for silicon and 0.0072 for polystyrene. For the DPC profiles the rmse are 0.015 and 0.098, for silicon and polystyrene, respectively. The main contribution to the rmse for both absorption and DPC originate from the cylinder borders, where the theoretical DPC signal diverges. The divergence of the DPC signal means that the phase shift, i.e. the local shift of the interference fringes is largest at the cylinder border. This can lead to a discretization issue when an interference fringe is shifted entirely outside the pixel without another one reentering from the other side, which can cause artificial absorption signals not accounted for by the theoretical signal based on Beer-Lambert law.

### Deposited energy

In order to demonstrate the feasibility of the simulation framework to explicitly simulate scattering related quantities the deposited energy is simulated and compared to the deposited energy obtained by conventional MC particle transport provided by a modified tutor2pp EGSnrc user code that doesn’t include diffractive effects at gratings or interfaces. For the sake of computation time, electron transport is not performed, instead all energy transferred to charged particles is assumed to be deposited locally. For maximal impact of Fourier splitting on deposited energy, the simulation setup in Fig. 5 is inverted with the sample after the G1 grating with a distance of 0.9 mm between the cylinder axis and the waver. Simulations with Fourier splitting were performed on three slightly different EGSnrc geometries, once without the periodic grating structure, in this case attenuation is modeled by transmission functions, which we refer to as implicit grating case. Another two simulations are performed with the periodic structure present as an EGSnrc geometry to model interactions in the grating slits. In order to combine transmission function based Fourier splitting with a grating geometry filled with media, a new EGSnrc medium equivalent to silicon in terms of cross sections but equivalent to air in terms of phase shift is generated to prevent errors in phase shift at the grating. For cases containing the EGSnrc grating geometry, the periodic structure was once placed right before and once after the splitting plane. The simulations are performed with $5\times 10^8$ histories and Fourier splitting with $N_{G1} = 5$ leading to a reported statistical uncertainty (standard deviation) of less than $2\times 10^{-3}$. The resulting deposited energies for each structure including energy leaving through the detector plane and energy leaving anywhere else are displayed in Table 2.

The largest relative difference of 2.6% for the deposited energy occurs for the simulation with an implicit grating, i.e. when attenuation is performed by the optics component via Beer-Lambert law. However, the absolute error remains small and is mainly affecting energy deposition inside the grating, which for implicit gratings is calculated as the left over energy due to overall reduction of the statistical weights at the grating (26). This is improved in the cases containing an EGSnrc grating geometry, for which the maximal relative error of 1.2% occurs for the energy leaving the geometry through the sides. Since diffraction at the grating and refraction at the cylinder surfaces are expected to broaden the beam, small differences compared to conventional tutor2pp simulations are expected. Hence, the results in Table 2 show that the presented algorithm preserves MC deposited energy features.

**Table 2:** Deposited energy in percent for the different structures.

Structure	tutor2pp	No G1 geom	Rel. error	G1 after	Rel. error	G1 before	Rel. error
leave at D	52.275	52.272	− 0.006	52.239	− 0.069	52.238	− 0.071
leave elsewhere	3.363	3.346	− 0.506	3.403	1.190	3.400	1.160
surrounding air	0.095	0.095	0.000	0.094	− 1.050	0.095	0.000
wafer	21.404	21.397	− 0.033	21.403	− 0.005	21.402	− 0.010
G1	0.932	0.956	2.580	0.931	− 0.107	0.931	− 0.107
Si cylinder	21.041	21.043	0.010	21.040	− 0.005	21.042	0.005
Polystyrene cylinder	0.889	0.890	0.112	0.889	0.000	0.890	0.112

The reference case simulated with the adapted tutor2pp in the first column is followed by the three test cases and the relative error compared to the tutor2pp results in percent. The three test cases are ’no G1 geom’ where G1 attenuation is simulated by the transmission function, ’G1 after’ and ’G1 before’ where the periodic structure is present as MC geometry after and before the splitting, respectively. The MC reported standard deviation is below $2\times 10^{-3}$ for all deposited energies.

### Visibility in a Talbot–Lau laboratory setup

**Figure 7:**
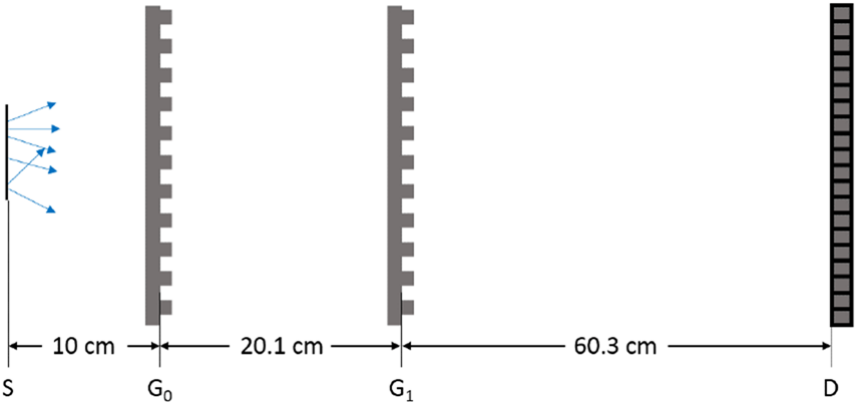
The table-top X-ray phase contrast micro computed tomography system simulated without the analyzer-grating G2. Initial positions of the particles are generated by the source S are normally distributed in x-direction with a FWHM of 10 $\upmu$m. The initial directions are collimated on the detector D. The inter grating distances are 20.1 cm and 60.3 cm, the grating periods are 1.0 $\upmu$m, 1.5 $\upmu$m, and 3.0 $\upmu$m for G0, G1 and G2, respectively. The duty cycle of all gratings is 0.5.

After the successful simulation of interference patterns in academic setups, the simulation of a GI setup for pathology samples Fig. 7 serves as a proof of principle demonstrating the potential of the algorithm to model GI setups with incoherent sources. The table-top X-ray phase contrast micro computed tomography system^33^ is composed of three 0.5 duty cycle gratings G0, G1, and G2 with the corresponding periods $p_0=1\,\upmu$m, $p_1=1.5\,\upmu$m, and $p_2=3\,\upmu$m. The inter grating distances are 20.1 cm between G0 and G1 and 60.3 cm between G1 and G2.

The simulation of 0.5 cm field of view (FOV) around the optical axis is done with three adaptations: (1) The analyzer grating in front of the detector is not simulated, instead the detector directly records the interference patterns. Phase stepping is simulated in a post processing step rebinning the data into $75\,\upmu$m sized pixels and using binary masks. (2) The spatial and directional dependence of the source, modeled by $P_S({\mathbf{r}}_S, {\mathbf{k}}_S)$ in Eqs. (6) and (28) is set to a collimated Gaussian shaped line source with a FWHM of 10 $\upmu$m that emits photons isotropically. (3) For the source spectrum, i.e., the energy dependence of $P_S({\mathbf{k}}_S, {\mathbf{r}}_S)$, a filtered idealized 40 keV triangle X-ray spectrum with a peak around the design energy of 19 keV is used.

Two simulations, one with the grating in place and a reference simulation without gratings, of $2.5\times 10^5$ incoherent particle histories were performed. At the source grating each path was split into $1.2\times 10^6$ paths with *Q*-values between $\pm 2\frac{2\pi }{a}$ according to (22) and splitting into Fourier coefficients between $N_{G1}=\pm 9$ (25, 26) at the phase grating. The simulations were split into 10 parallel tasks using both cores to reduce overall waiting time, each taking roughly 19 h of computation time.

**Figure 8:**
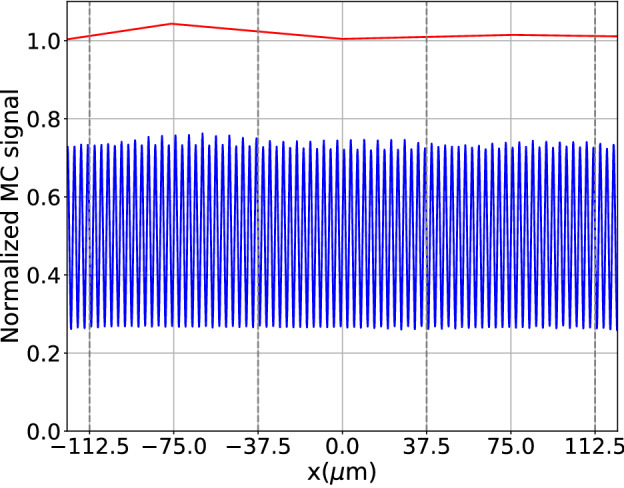
MC generated signals for the laboratory system limited to two pixels of 75 $\upmu$m (indicated by gray lines): rebinned signals with gratings (blue) and without (red). Both signals are normalized by the mean signal without gratings. The MC signal has the theoretically expected and experimentally verified periodicity of 3 $\upmu$m.

The recorded MC signals for the three central detector pixels shown in Fig. 8 are normalized with the average of the signal without gratings. The total signal over the full FOV of 0.5 cm is reduced by 49.15% when gratings are present. Classically expected, assuming a perfectly absorbing source grating, a $25\,\upmu$m thick silicon phase grating (both with a duty cycle of 0.5), and orthogonal illumination, is a reduction of the intensity in the center by 49.5%. The difference of 0.4% can easily be explained by the more complex geometry and by a slight broadening of the beam due to the splitting at the gratings, as the detector doesn’t cover the full width of the beam. The correct normalization of the interference pattern strongly suggests the compatibility with the estimation of scattering related quantities. The simulated signal has the theoretically expected and experimentally verified periodicity of 3 $\upmu$m. Neglecting the two outermost pixels on each side to limit boundary effects, the calculated fringe visibility *v* is in the range of $v_\text {min} = 0.275$ and $v_\text {max} = 0.288$ with an average visibility of 0.282. This is higher than the experimentally reported visibility around 0.195 in the center and the high visibility areas with a visibility around 0.23. However, an overestimation is expected due to the employed simplifications like the source shape and spectrum, imperfect experimental conditions (e.g. small grating tilts), idealized gratings, e.g. leakage through the absorbing parts of the source and analyzer gratings and detector response. For instance, for monochromatic beams the impact of the leakage though an analyzer grating with duty cycle 0.5 can be estimated classically with a visibility correction factor

1 $$\begin{aligned} c_\text {cor}(\lambda ) = \frac{T_\text {SI}(\lambda )-T_\text {AU} (\lambda )}{ T_\text {SI}(\lambda )-T_\text {AU} (\lambda )} \end{aligned}$$

taking into account the transmission through the gold $T_\text {AU}(\lambda )$ and the silicon sections $T_\text {SI}(\lambda )$ of the grating. For a 30 $\upmu$ thick analyzer grating^33^ at the mean energy of the simulation spectrum (21 keV) the correction factor $c_\text {cor}(21\, \text {keV}) = 0.96$ and decreases for higher energies. Although calculated for a monochromatic beam, this effect can explain a few percent overestimation of the visibility when compared to experiment. Additionally, the transmission trough the absorbing iridium sections of the source grating ($\sim 1.5$% at 21 keV) further reduces the visibility.

## Conclusion

The newly proposed and implemented MC algorithm is feasible for the simulation interference phenomena from microscopic to macroscopic scales as well as for the simulation of scattering effects such as deposited energy. The c++ object oriented design allows for extensions such as the introduced variance reduction techniques for gratings, which can achieve significant speedup in specific setups compared to the more general splitting approaches such as Huygens splitting. The far field like approximation for the variation reduction techniques and the simplified source model are supported by the successful simulation of interference patterns in academic setups and the visibility in a table-top X-ray phase contrast system. Furthermore, with the simulation of a mm-sized field of view on a single core with acceptable simulation times and the straightforward parallelization, the algorithm has shown its potential for scalability to larger volumes required for the simulation of clinical prototypes. The simulated intensity reduction in the lab setup and the deposited energy simulations assure that no intensity or energy is created or destroyed by the gratings or the interference effects during simulation. With significant improvement in simulation time, Fourier splitting has great potential for future extensions to bent grating setups, where the effects of grating aspect ratios are minimized. For flat gratings the piecewise constant transmission function approach has limitations, e.g., for higher energies that require high aspect ratio gratings. However, the thin grating assumption and the transmission functions are not essential assumptions for the presented framework, which offers alternative options for transport through optical components. Future optimizations of the transport parameters for the path splitting carry the potential for a better trade-off between accuracy and computation time, especially for Fourier splitting.

## Methods

### Expected detector signal

The expected detector signal in a GI imaging device is obtained based on fundamental quantum mechanical concepts for photon propagation, creation - or photon sources, and scattering^34^ and classical approximations.

#### Propagation

Photon transport from ${\mathbf{r}}$ to ${\mathbf{r}}^\prime$ in vacuum is modeled through the single photon amplitude in real space

2 $$\begin{aligned} A^k({\mathbf{r}}, {\mathbf{r}}^\prime ) = \frac{e^{i k \left| {\mathbf{r}} - {\mathbf{r}}^\prime \right| }}{\left| {\mathbf{r}} - {\mathbf{r}}^\prime \right| } \end{aligned}$$

for a photon of frequency $\omega = c k$, where *c* denotes the speed of light. At this point it is worth noting that all expressions are only to be understood up to a normalization constant, since normalization will be enforced during MC run time. The sum over indistinguishable paths from source to detector of a single photon is the origin of interference phenomena^34,35^ and the use of the amplitude (2) was shown to be equivalent^36^ to conventional optics^16^ and statistical optics^37^ for simple diffraction experiments recording only the field intensity^38^.

When optical components such as gratings or material interfaces are introduced between the source and the detector in order to generate interference patterns, the classical approximation of the photon traveling in straight lines inside homogeneous isotropic media, that is used in conventional MC, breaks down. Instead, the amplitudes of all indistinguishable paths to arrive at the detector have to be summed. For the purpose of X-ray GI imaging systems, it is sufficient to restrict the sum over indistinguishable paths to a subset of piecewise straight paths crossing smooth interfaces $G_1,\dots ,G_N$ covering the full field of view. According to the rules of quantum mechanics of adding amplitudes^34,39,40^ for alternative events and multiplying amplitudes for consecutive events the overall amplitude $A_G^k({\mathbf{r}},{\mathbf{r}}^\prime )$ becomes a sum over the smooth surfaces $G_j$ between source and detector

3 $$\begin{aligned} A_G^k({\mathbf{r}},{\mathbf{r}}^\prime ) = \int _{G_N}dG_N \cdots \int _{G_1}dG_1 A^k({\mathbf{r}},{\mathbf{r}}_{G_N})A^k({\mathbf{r}}_{G_N}, {\mathbf{r}}_{G_{N-1}})\cdots A^k({\mathbf{r}}_{G_{1}},{\mathbf{r}}^\prime ), \end{aligned}$$

with ${\mathbf{r}}_{G_j}\in G_j$ and the surface element $dG_j$ of the $j^\text {th}$ interface $G_{j}$. As in classical wave theory contributions to the integral from outside the field of view are safely neglected.

#### Sources

In classical optics the initial field emitted by a source is usually characterized by its transverse or longitudinal coherence length^41^. For simplicity we start with transverse and longitudinal coherent monochromatic spherical and plane waves and expand to X-ray tubes, which are assumed to have practically zero transverse and longitudinal coherence.

In the case of the coherent sources it is assumed that photon creation in the source S is associated with an amplitude $\psi ({\mathbf{r}}^\prime ,S,k)$. Using the rules for combining amplitudes again, the overall amplitude (3) has to be multiplied by $\psi ({\mathbf{r}}^\prime ,S,k)$ and integrated over the source volume $V_S$ (or area). The probability $P_p(j,S,k)$ for a coherent source S to find a photon in a detector pixel *j* with volume $V_j$ of a pixel detector is given by the integration over the absolute value squared of the overall amplitude

4 $$\begin{aligned} P_p(j,S,k) = \int _{V_j}{d^3 r\left| \int _{V_S}{d^3r^\prime A_G^k({\mathbf{r}},{\mathbf{r}}^\prime )\psi ({\mathbf{r}}^\prime ,S,k)}\right| ^2}. \end{aligned}$$

For efficient modeling of more complex photon sources, such as X-ray tubes, additional assumptions and simplifications on primary photon creation are made: (1) The source creates disentangled photons, i.e., the interference patterns originate only from the optical components along the photon path, which is supported by the intensity-independence of the experimentally observed interference patterns. (2) Different post-scattering states of a bremsstrahlung event do not interfere on the detector, since they correspond to different final states of the whole system. (3) A photon created by a scattering event in the anode of an X-ray tube is assumed to behave like a wave-packet $\psi _{{\mathbf{k}}_S{\mathbf{r}}_S}({\mathbf{r}}^\prime )$ initially localized within a small volume around ${\mathbf{r}}_S$ moving along ${\mathbf{k}}_S$. Therefore, by setting $\psi ({\mathbf{r}}^\prime ,S,k) = \psi _{{\mathbf{k}}_S{\mathbf{r}}_S}({\mathbf{r}}^\prime )$ individual photons^42^ are propagated and the probability to find the photon inside pixel *j* is given by

5 $$\begin{aligned} P_{{\mathbf{k}}_S{\mathbf{r}}_S}(j,S)=\int d^3r\left| \int d^3 r^\prime A_G^{k_S}({\mathbf{r}},{\mathbf{r}}^\prime )\psi _{{\mathbf{k}}_S{\mathbf{r}}_S}({\mathbf{r}}^\prime )\right| ^2 \end{aligned}$$

with $k_S=\left| {\mathbf{k}}_S\right|$. However, in contrast to the perfectly coherent sources Eq. (4) the contributions of many photons labeled by the wave-vector ${\mathbf{k}}_S$ and position ${\mathbf{r}}_S$ have to be summed

6 $$\begin{aligned} P_p(j,S) = \sum _{{\mathbf{k}}_S,{\mathbf{r}}_S} P_S({\mathbf{k}}_S,{\mathbf{r}}_S) P_{{\mathbf{k}}_S{\mathbf{r}}_S}(j,S) = \sum _{k_S}P_p(j,S,k_S), \end{aligned}$$

where $P_S({\mathbf{k}}_S,{\mathbf{r}}_S)$ is the probability to create a primary photon in an area around ${\mathbf{r}}_S$ moving along ${\mathbf{k}}_S$. The probability $P_S({\mathbf{k}}_S,{\mathbf{r}}_S)$ is modeled classically either by conventional MC particle transport or by sampling directly from a source spectrum, which can be position and direction dependent. In practice it allows the use of a spectrum and a source spot size to model incoherent polychromatic sources. It should be noted at this point that with a suitable choice of $\psi _{{\mathbf{k}}_S{\mathbf{r}}_S}$ sources with non-zero coherence length can also be modeled.

#### Scattering

Performing a full path integral calculation for a macroscopic system would require to sum over a prohibitive number of amplitudes, especially when a medium is present. Therefore, additional simplifications are necessary in order to achieve acceptable simulation times. Revisiting the rules for calculating amplitudes and probabilities it is important to notice that for distinguishable paths, e.g., through an incoherent scattering event inside a medium, the corresponding probabilities are added rather than the amplitudes as in Eq. (3). Hence, for a monochromatic source the probability $P_{\text {p}}(j,S,k)$ to find a primary photon of energy $E_k = \hbar c k$ in a pixel *j* of a detector and the corresponding probability $P_{\text {sc}}(j,S,k^\prime , k)$ to find a scattered photon with $E_{k^\prime } < E_k$ can be used to write the expected detector signal ${\langle D(S,k) \rangle _{j} }$ for monochromatic sources as the sum of primary and secondary (or scattered) contributions

7 $$\begin{aligned} {\langle D(S,k) \rangle _{j} }= s(k)P_p(j,S,k)+\sum _{k^\prime }s(k^\prime )P_{\text {sc}}(j,S,k^\prime ,k), \end{aligned}$$

assuming an ideal detector that generates a signal *s*(*k*) for each photon of energy $E_k$. The same principle of adding probabilities for primary and secondary signals applies in the incoherent case in a photon wise manner (compare Eqs. (4), (5), and (6)), i.e. summing over the contributions from different initial positions and wave-vectors which can be expressed as a sum over $k_S$ as in Eq. (7)

8 $$\begin{aligned} {\langle D(S) \rangle _{j} }=&\sum _{{k}_S} \langle D(S,{k}_S) \rangle _{j}. \end{aligned}$$

One goal of a GI MC is to estimate the expected detector signal in the presence of samples and optical elements for coherent and incoherent sources. This is achieved with a ray-tracing algorithm for the calculation of $P_p(j,S,k)$. Explicit scattering events are reintroduced in a second step into the ray-tracing algorithm modeling $P_{\text {sc}}(j,S,k^\prime ,k)$.

### Ray-tracing algorithm

For the calculation of the primary signal $P_p(j,S,E)$ explicit scattering events are not of interest. Instead, only the decrease of the amplitude for the photons to travel through the medium without a scattering event to happen is needed. Therefore, for large distances the transition amplitude is modified by a reduction of the norm and an additional phase shift, expressed with the complex refractive index $n_m$ of the medium *m*, which for X-rays often is expressed as $n_m= 1-\delta _m + i\beta _m$. Therefore, in an infinite medium the transition amplitude given in Eq. (2) is modified by the replacement $k\rightarrow n_m k$ (suppressing explicit *k*-dependence of $n_m$), which results in the amplitude inside a medium *m*

9 $$\begin{aligned} A_{m}^k({\mathbf{r}},{\mathbf{r}}^\prime ) = \frac{e^{i n_m k \left| {\mathbf{r}} - {\mathbf{r}}^\prime \right| }}{\left| {\mathbf{r}} - {\mathbf{r}}^\prime \right| } \end{aligned}$$

that replaces $A^k({\mathbf{r}},{\mathbf{r}}^\prime )$ in all calculations.

#### Sources

Modeling of different types of sources depends on the photon creation amplitude $\psi$. Thereby, Eqs. (4) and (5) serve as starting point for the development of the basic ray-tracing algorithm, which, due to the similar form of Eqs. (4) and (5) can be used for incoherent sources in a photon wise manner (6) with only few adaptations. For validation purposes it is worth to consider simple coherent sources such as plane waves and point sources.

The simplest source *S* is a plane wave, obtained by setting $\psi ({\mathbf{r}}^\prime ,S,k) = 1$ for positions ${\mathbf{r}}^\prime$ on a source plane $S_0$. The probability $P_\text {p}(j,\text {pw},k)$, where ’pw’ stands for ’plane wave’, for a detector pixel *j* is then expressed through the integral over the source plane

10 $$\begin{aligned} P_{\text {p}} (j,\text {pw},k) = \int _{V_j}d^3r \left| \int _{S_0} dS_0 A_G^{k}({\mathbf{r}},{\mathbf{r}}^\prime )\right| ^2 \end{aligned}$$

with the surface area element $dS_0$.

In an analogue way the probability $P_{\text {p}} (j,\text {sw},k)$, for a spherical wave (’sw’) is obtained by the identification of $\psi ({\mathbf{r}}^\prime ,\text {sw},k)$ with a delta function in three dimensions $\delta ^{(3)}( {\mathbf{r}}^\prime -{\mathbf{r}}_S)$ resulting in

11 $$\begin{aligned} P_{\text {p}} (j,\text {sw},k) = \int _{V_j}d^3r \left| A_G^{k}({\mathbf{r}},{\mathbf{r}}_S)\right| ^2. \end{aligned}$$

The assumption that incoherent sources, in the following abbreviated as ’ic’, create disentangled photons localized within a small source spot labeled by ${\mathbf{r}}_S$ allowed to express $P_p(j,\text {ic},k)$ (6) as a summation over the macroscopic extension of the source. However, the amplitude for photon creation $\psi _{{\mathbf{k}}_S{\mathbf{r}}_S}$ required for individual photon transport (5) is unknown. For the sake of computation time it is assumed that the initial photon can be approximated classically with sharply defined position and momentum similar to conventional MC particle transport, which results in

12 $$\begin{aligned} P_{{\mathbf{k}}_S{\mathbf{r}}_S}(j,S) = \int _{V_j} d^3r\left| A_G^{k_S}({\mathbf{r}},{\mathbf{r}}_S)\right| ^2. \end{aligned}$$

As a consequence, only the central mode of the wave packet is considered and integrals of the form (3) are calculated with plane waves instead of wave packets. The missing falloff of the wave packet is addressed by an artificial limitation of the integration area to the close vicinity of the intersection of the classical path with the surface *G* in Eq. (3).

The different forms of Eqs. (10) and (11) compared to (6), impact the calculation of the detector signal in the ray-tracing algorithm. In the absence of optical components and interfaces photons emitted by incoherent sources as from Eq. (12) are simulated by starting a single trajectory at ${\mathbf{r}}_S$ in the direction of ${\mathbf{k}}_S$ (eventually generated according to a source distribution and a spectrum), which continues in a straight line until it is terminated when it reaches the detector or leaves the simulation geometry. This translates one to one to a particle history in conventional MC particle transport, apart from explicit scattering events, which will be addressed later. During the straight trajectories the algorithm keeps track of the amplitude $A^{{k}_S}_m({\mathbf{r}},{\mathbf{r}}_S)$. After a history has been terminated the detector signal is computed according to (12) and then added to the total detector signal (6) before a new photon history is started. Thereby different photons are treated independently each creating a detector signal (if the detector is hit).

For coherent sources several straight line trajectories (or histories) with different initial positions (10) or initial directions (11), respectively, are required for the simulation of a single particle. Each history represents an indistinguishable photon path, hence, the corresponding amplitudes have to be added on the detector as indicated in (10) and (11). This can be seen as an attempt to model the uncertainty in position or momentum in the ray-tracing algorithm. In both cases the due to the random nature of the path generation it is unfeasible to guarantee that paths have the same endpoints. For the summation over the amplitudes the detector is discretized into small data points. Amplitudes of photon paths that end in the same data point are added coherently. The summation over the pixel volume $V_j$ is performed after the simulation by adding the signals of all data points inside the detector pixel.

#### Optical components

##### Huygens splitting

When interfaces or optical components are present the amplitudes in Eqs. (10), (11), and (12) contain several integrals of the form (3) over surfaces $G1,\dots , G_N$. In the ray-tracing algorithm this translates into multiple particle trajectories that cross the interfaces or optical components, e.g. gratings at different locations, which can be solved by the introduction of a uniform path splitting, which imitates the Huygens-Fresnel principle as it has been done in previous approaches^26,27^. In this work so called Huygens splitting, is implemented as a planar optics component that splits every incoming primary path into $N_{\text{ G }1}$ outgoing paths with equal starting positions, statistical weights, phases and norms. The paths are distributed uniformly on an arc of a user defined angular range with the intention to ensure full coverage of the field of view, while reducing the number of paths as that are not propagated towards the detector.

#### Variance reduction

In MC particle transport variance reduction techniques such as range rejection or interaction forcing^43^ are widely used practices to reduce computation time. In the same spirit this section introduces variance reduction methods for the transport of the paths in a MC ray-tracing algorithm. Instead of including as many paths as possible in the ray-tracing algorithm, rules for the “important” paths that have to be considered can be derived. For instance, whenever a photon encounters an interface there is an amplitude for transmission and reflection. However, apart from total reflection at interfaces reflection is not taken into account as it is assumed to not be of any significance for the generation of interference patterns in X-ray GI, which could already be seen as variance reduction. In the following two variance reduction techniques—referred to as Fourier splitting—for the propagation through source and phase gratings are developed in the corresponding sections Absorption gratings and Phase gratings. In the Medium interfaces section Snell’s law is introduced, as in previous simulation approaches, as generic method for reduction of simulation time at medium interfaces.

##### Fourier splitting

For the purpose of Talbot-Lau interferometry, it is often sufficient to approximate the optics components as infinitesimally thin, which allows to define them as complex valued transmission functions

13 $$\begin{aligned} \tau _{\text {G}}(x,k) = {\left\{ \begin{array}{ll} \tau _a(k),\,x\in \left[ jp_\text {G},jp_\text {G} + a\right) \\ \tau _b(k),\,x\in \left[ jp_\text {G}+a,(j+1)p_\text {G}\right) , \end{array}\right. } \end{aligned}$$

where $j\in {\mathbb {Z}}$ labels the grating period, $\tau _a(k)$ and $\tau _b(k)\in {\mathbb {C}}$ correspond to the (energy dependent) transmission function values in the two grating sections of length a and b, respectively, and $p_\text {G}=a+b$ equals the grating period. The transmission function formulation reduces the number of integrals by one and replaces the one transition amplitude in equation (3), resulting in a simplified expression for the overall transition amplitude $A_G^{k}({\mathbf{r}},{\mathbf{r}}_S)$ in Eqs. (10), (11), and (12) in the presence single grating *G* between two regions filled with media *A* and *B*

14 $$\begin{aligned} A_G^k({\mathbf{r}},{\mathbf{r}}^\prime ) = \int _{G}dG A_B^k({\mathbf{r}},{\mathbf{r}}_G)\tau _G({\mathbf{r}}_G,k)A_A^k({\mathbf{r}}_G,{\mathbf{r}}^\prime ) \end{aligned}$$

for ${\mathbf{r}}_G\in G$. In an attempt to reduce the number of paths required to propagate a single photon the contribution $A_{U({\mathbf{r}}_j)}^k({\mathbf{r}},{\mathbf{r}}^\prime )$ of a small neighborhood $U({\mathbf{r}}_j) \subset G$ around a point ${\mathbf{r}}_j \in G$ to the amplitude $A_G^k$ at ${\mathbf{r}}$ is considered. When modeling a flat grating GI setup the surface *G* is considered to be parallel to the xy-plane (with the main propagation direction along the z-axis) which allows to parameterize the neighborhood $U({\mathbf{r}}_j)$ as

15 $$\begin{aligned} {\mathbf{r}}_G = {\mathbf{r}}_j + {\varvec{\eta }}\,\forall {\mathbf{r}}_G\in U({\mathbf{r}}_j) \end{aligned}$$

with $\eta = (\eta _x,\eta _y,0)^T$ and $\eta _x\in (\eta _x^\text {min}, \eta _x^\text {max})$ and $\eta _y\in (\eta _y^\text {min}, \eta _y^\text {max})$. For sufficiently small neighborhoods $U({\mathbf{r}}_j)$ it is possible to use^16^

16 $$\begin{aligned} \left| {\mathbf{r}} - {\mathbf{r}}_G \right| ^{-1}\approx \left| {\mathbf{r}} - {\mathbf{r}}_j \right| ^{-1} \end{aligned}$$

and

17 $$\begin{aligned} \left| {\mathbf{r}} - {\mathbf{r}}_G \right| \approx \left| {\mathbf{r}} - {\mathbf{r}}_j \right| - \frac{{\mathbf{r}}-{\mathbf{r}}_j}{\left| {\mathbf{r}} - {\mathbf{r}}_j \right| }{\varvec{\eta }} \end{aligned}$$

to approximate the amplitude in Eq. (9) with a linear phase term.

Neglecting the imaginary part of the refractive index, and introducing the difference of the wave vectors before and after *G*

18 $$\begin{aligned} \mathbf{Q} = (Q_x,Q_y,Q_z)^T := -n_B k \frac{{\mathbf{r}}-{\mathbf{r}}_j}{\left| {\mathbf{r}} - {\mathbf{r}}_j \right| } + n_A k \frac{{\mathbf{r}}_j-{\mathbf{r}}_S}{\left| {\mathbf{r}}_G - {\mathbf{r}}_S \right| } \equiv -n_B{\mathbf{k}} + n_A{\mathbf{k}}_S \end{aligned}$$

allows to rewrite the contribution $A_{U({\mathbf{r}}_j)}^k({\mathbf{r}},{\mathbf{r}}^\prime )$ in the convenient form

19 $$\begin{aligned} A_{U({\mathbf{r}}_j)}^k({\mathbf{r}},{\mathbf{r}}^\prime ) \approx A_{B}^{k}({\mathbf{r}},{\mathbf{r}}_j)\text {exp}\left[ -i (Q_x x_j + Q_y y_j)\right] {\tilde{\tau }}_{G}(Q_x,Q_y, k)A_A^{k}({\mathbf{r}}_j,{\mathbf{r}}^\prime ), \end{aligned}$$

where ${\tilde{\tau }}_{G}(Q_x,Q_y)$ denotes the Fourier integral over a restricted area $U({\mathbf{r}}_j)$

20 $$\begin{aligned} {\tilde{\tau }}_G(Q_x,Q_y) = \int _{U({\mathbf{r}}_j))}dx_G dy_G\,\text {exp}\left( i (Q_x x_G + Q_y y_G )\right) \tau _G({\mathbf{r}}_G, k) \end{aligned}$$

with ${\mathbf{r}}_G = (x_G,y_G,z_G)$ and ${\mathbf{r}}_j = (x_j,y_j,z_j)$. In the following Eqs. (19) and (20) serve as basis for the development of variance reduction techniques for photon transport through gratings. The resulting transport rules are significantly faster than the implementation of uniform splitting to imitate Huygens principle.

##### Absorbing gratings

Absorption gratings, labeled as G0, are assumed to be $p_{G0}$ periodic binary gratings with $\tau _a=1$ and $\tau _b=0$. Because source gratings in GI are placed close to the source and have periods much bigger than the X-ray wavelengths, the wave-packets that model the primary photon are assumed to be localized within one grating slit. Therefore, the photon is discarded if the classical photon path hits an absorbing section ($\tau _\text {G0}(x,k) = 0$) of the grating and transmitted otherwise. Similarly, for coherent sources all paths that intersect the grating at an absorbing section are discarded. The natural limitation $U({\mathbf{r}}_j)$ of the spatial integral is one grating slit ($\tau _\text {G0}(x,k)=1$) labeled by *j*, which turns Eq. (20) into the Fourier transform of the grating slit

21 $$\begin{aligned} {\tilde{\tau }}_{\text {G0}}(Q_x,Q_y) =\, \text {exp}\left[ i Q_x(j p_{G0}+a/2)\right] 2\frac{\sin \left( \frac{Q_x a}{2}\right) }{Q_x} 2 \pi \delta (Q_y). \end{aligned}$$

The delta function assures that there is only a contribution to the amplitude Eq. (19) if the paths satisfies $Q_y=0$. However, as expected there is a non-zero contribution to the amplitude for different x-directions. This is implemented by splitting the incoming straight line path of a photon into many paths with directions ${\mathbf{k}}_j$, each corresponding to a $\mathbf{Q}_j$, with a randomly chosen *x*-component $Q_{jx}$ and a *z*-component that ensures energy conservation $\left| {\mathbf{k}}_j\right| =k$. Each path is weighted by a complex weight

22 $$\begin{aligned} z_j = N\text {exp}\left[ i Q_{jx}(j p_{G0}+a/2-x_j)\right] \frac{\sin \left( \frac{Q_{jx} a}{2}\right) }{Q_{jx}/2}, \end{aligned}$$

where *N* assures normalization $\sum _j{\left| z_j\right| ^2}=1$. Momentum conservation is assured by always including the paths corresponding to $\pm Q_{jx}$ in the splitting, which ensures that the grating doesn’t introduce a random drift into positive or negative x-direction.

##### Phase gratings

Analogous to absorption gratings the phase shift introduced by phase gratings, denoted by G1, is described by a piece-wise constant $p_{\text {G1}}$ periodic transmission function $\tau _{\text {G1}}(x, k)$ of the form of Eq. (13). In GI phase gratings are used after a source grating G0 or with a coherent source. Therefore, the limitation of the integration area $U({\mathbf{r}}_j)$ is extended to several grating periods, assuming plane wave illumination. The calculation of Eqs. (19) and (20) for phase gratings, is done by expressing the grating transmission function as a Fourier series

23 $$\begin{aligned} \tau _{\text {G1}}(x,k) = \sum _{n=-N_{G1}}^{N_{G1}}{{\hat{\tau }}_{\text {G1}}(n,k)\,\text {exp}\left( -i\frac{2\pi n}{p_{\text {G1}}}x\right) } \end{aligned}$$

restricted to $2N_{G1} + 1$ Fourier coefficients, with

24 $$\begin{aligned} {\hat{\tau }}_{\text {G1}}(n,k)=\frac{1}{p_{\text {G1}}}\int _{0}^{p_{\text {G1}}}{\tau _{\text {G1}}(x,k)\,\text {exp}\left( i\frac{2\pi n}{p_{\text {G1}}}x\right) } \end{aligned}$$

in Eq. (20), which results in a discrete sum over Fourier coefficients for the transmission amplitude through phase gratings

25 $$\begin{aligned} A_{G1}^k({\mathbf{r}},{\mathbf{r}}_j) = \sum _{n=-N_{G1}}^{N_{G1}} {e^{in _B k\left| {\mathbf{r}}-{\mathbf{r}}_j\right| }{\hat{\tau }}_{\text {G1}}(n,k)}\,\text {exp}\left( -i\frac{2\pi n}{p_{\text {G1}}}x_1^{cl}\right) \delta \left( Q_x - \frac{2\pi n}{p_\text {G1}}\right) \delta (Q_y) e^{in _A k\left| {\mathbf{r}}_j-{\mathbf{r}}_S\right| }. \end{aligned}$$

This is implemented as a splitting procedure similar to source gratings with three differences. First, the directions changes $Q_x$ are not selected randomly but correspond to a finite set of augments $Q_{nx}=\frac{2\pi n}{p_{\text {G1}}}$ of the Fourier series. Second, apart from a position dependent phase term, all photon paths are transported through the grating independent of their intersection with the grating. Third, the weights applied to each path

26 $$\begin{aligned} z_n={\hat{\tau }}_{\text {G1}}(n,p_S)\text {exp}\left( -i\frac{2\pi n}{p_{\text {G1}}}x_1^{cl}\right) \end{aligned}$$

are normalized due to Parseval’s theorem. The splitting procedures for phase and absorption gratings have great potential to improve simulation time since they allow to restrict the transport to relevant paths and give the user a way to define a maximum angle to be considered in the simulation.

##### Medium Interfaces

Instead of splitting the paths at every medium interface, e.g. by using the implemented Huygens splitting, which would generate an unfeasible number of paths, Snell’s law is implemented as classical approximation. With the above notation (18) the corresponding path amplitude can be expressed as

27 $$\begin{aligned} A_{U({\mathbf{r}}_j)}^k({\mathbf{r}},{\mathbf{r}}^\prime ) = A_{B}^{k}({\mathbf{r}},{\mathbf{r}}_j)\tau \delta ({Q_x})\delta (Q_y) A_A^{k}({\mathbf{r}}_j,{\mathbf{r}}^\prime ). \end{aligned}$$

The delta functions ensure that there is only a non-zero contribution at positions ${\mathbf{r}}$ satisfying $Q_x = 0$ and $Q_y=0$ imposing two conditions on the direction change (18) of the path that are equivalent to Snell’s law. In this way classical behavior is established at interfaces. In the ray-tracing algorithm this is simulated by a piece-wise straight trajectory with a change of direction at the interface satisfying Snell’s law. Snell’s law is applied for any shape and size of interfaces in the ray-tracing algorithm. Additionally, in order to save simulation time only the transmitted part of the amplitude is considered during ray-tracing (setting $\tau =1$), except for situations where total reflection occurs, in which case $\tau \rightarrow \tau _R = -1$.

### Introduction of explicit scattering events

The remaining step for estimating the expected detector signal (7) or (8) is to account for explicit scattering events. For simplicity, only the single-scattering case is investigated. The probability to find a scattered photon of energy *E* in pixel *j* depends on the source type *S*. For incoherent sources this probability is approximated as

28 $$\begin{aligned} P_{\text {sc}}^{\text {ic}} (j,S,k_\text {sc}) = \int _{V_j}d^3r\sum _{{\mathbf{k}}_\text {sc}{} {\mathbf{r}}_{\text {sc}}} \sum _{{\mathbf{k}}_S{\mathbf{r}}_S} \left| A_m^{k_\text {sc}}({\mathbf{r}},{\mathbf{r}}_{\text {sc}} )\right| ^2 P_{\text {sc}}^{m}({\mathbf{k}}_{\text {sc}},{\mathbf{k}}_{S}) \left| A_m^{{k}_S}({\mathbf{r}}_{\text {sc}},{\mathbf{r}}_S)\right| ^2 P_S ({\mathbf{r}}_S,{\mathbf{k}}_S) \end{aligned}$$

where $P_{\text {sc}}^{m}({\mathbf{k}}_{\text {sc}},{\mathbf{k}}_{S})$ denotes the probability that a photon with energy $\hbar c \left| {\mathbf{k}}_S\right|$ moving in direction of ${\mathbf{k}}_S$ undergoes a scattering event resulting into a photon of energy $\hbar c \left| {\mathbf{k}}_\text {sc}\right|$ in direction of ${\mathbf{k}}_\text {sc}$ in medium *m* present at location ${\mathbf{r}}_{\text {sc}}$, which is given over the respective cross sections. Thereby interactions and the potentially resulting secondary particles are described in close analogy to incoherent sources, reflecting the similarity between the two processes. For coherent sources the above expression has to be adapted by dropping the summation over ${\mathbf{k}}_\text {sc}$ and ${\mathbf{r}}_{\text {sc}}$ and by setting $P_S ({\mathbf{r}}_S,\mathbf{p}_S)$ to 1.

**Figure 9:**
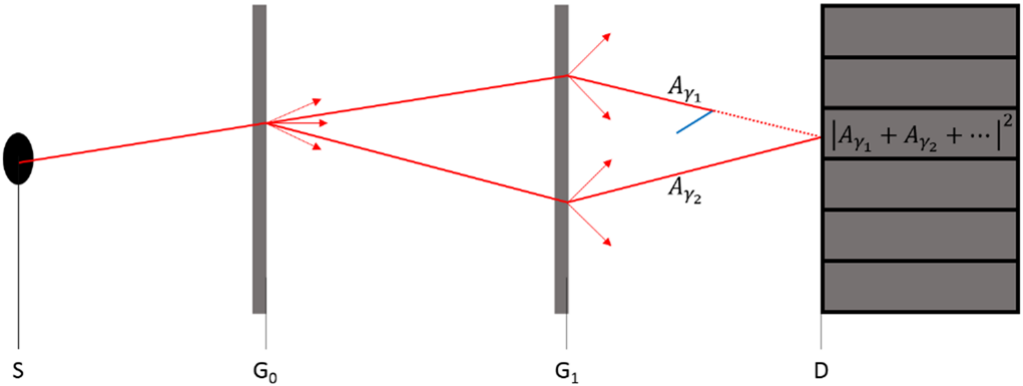
Visualization of the presented MC algorithm. Primary paths (red) generated by the source S are transported in straight lines until the crossing with material interfaces, gratings or a simulation boundary. At material interfaces path directions change according to Snell’s law, at gratings ($G_0$ and $G_1$) the paths fork into multiple paths (indicated by arrows) with complex weights given by Eqs. (22) and (26), respectively. Path amplitudes $A_{\gamma _j}$ that end in the same data point are summed and squared to calculate the detector signal (7). When EGSnrc is about to perform a scattering event, primary paths are continued in a ray-tracing mode, preventing further interactions along the path (red dashed). In the case of Rayleigh scattering the post-scattering particles are transported as primary paths, otherwise they are transported as secondary particles in EGSnrc MC (blue).

An implementation of Eq. (28) has to include the calculation of the probability to reach first the interaction location and then the detector, which would take an unfeasible number of paths to compute for each interaction. Instead, the primary signal is calculated with the introduced ray-tracing algorithm implemented within EGSnrc. During this calculation scattering events are allowed to happen along every photon path created by the ray-tracing algorithm. The probability for a scattering event along a straight path segment is governed by the Beer-Lambert law as in conventional MC, which is handled by EGSnrc. Before a scattering event is performed by EGSnrc the primary path is propagated in a pure ray-tracing mode disabling explicit scattering events in order to prevent an overestimation of scattering related quantities and to ensure that no primary path for the calculation of the interference pattern is lost. After the pure ray-tracing of the primary path EGSnrc performs the scattering event adding potential secondary particles to the particle stack. Similar to MC particle splitting, the weights of secondary particles are multiplied by a $\left| z_j\right| ^2$ factor, e.g. (26), if they occur on a path split by a grating, which follows from replacing $A_m^{{k}_S}({\mathbf{r}},{\mathbf{r}}_S)$ by $A_{G0}^{k_S}({\mathbf{r}},{\mathbf{r}}_S)$, $A_{G1}^{k_s}({\mathbf{r}},{\mathbf{r}}_S)$, or $A_{G1}^{k_S}({\mathbf{r}},{\mathbf{r}}')A_{G0}^{k_S}({\mathbf{r}}',{\mathbf{r}}_S)$ in Eq. (28). Any further propagation of secondary particles, with the exception of Rayleigh scattering, is assumed to produce no additional interference and, hence, is simulated with conventional EGSnrc MC particle transport without any splitting at the gratings. After a Rayleigh scattering event, however, the corresponding entry on the EGSnrc particle stack is handled as a primary path, including further splitting at gratings and its contribution to the primary signal. To limit the impact of Rayleigh scattering on computation time the number of such coherent scattering events is currently restricted to one per path. An illustration of the full GI MC is given in Fig. 9.

## Data Availability

The data used for the findings reported in this work can be generated with the example user codes and the corresponding input files provided on GitHub.

## Code availability

All code used to generate the results is provided on GitHub (https://zenodo.org/badge/latestdoi/454104492) under the GNU Affero General Public Licence.
